# Patent Medicines

**Published:** 1874-10

**Authors:** M. S. Bidwell


### PATENT MEDICINES.

M. S. BIDWELL.

In the Bistoury for April an attempt was made to answer the question—"Why are these humbugs so universally used?" We now propose to consider the cognate question. "Why should they not be used?" The first and most obvious reason is, that they are essentially of a fraudulent nature. Secrecy and deception naturally go hand-in-hand, and though the former does not necessarily lead to the latter, it does offer temptations too strong for the average nostrum-maker to resist. To take first the very lowest ground, as a mere matter of dollars and cents, they are usually a fraud. The manufacturer, entrenched behind the double barrier of secrecy and proprietary rights, and employing all the weapons of advertisement, does not hesitate to charge for his medicines in proportion to the size of the bottle, or the impudence of his claims, rather than according to the cost or value of his remedy. Two familiar instances out of many that might be adduced, will illustrate this point. Swain's Panacea, composed of a quart of molasses and water, with a few grains of corrosive sublimate, retails for two dollars. A pint of small beer, with a few cents' worth of aloes, sells for a dollar, under the guise of a "Temperance medicine" as Vinegar Bitters.

Again, they are always fraudulent in the claims they put forth. This is so notorious as to require no argument, but merely to notice that this is the natural result of vigorous and unscrupulous advertising. Let any one undertake to actively "puff" any article whatever, and he will soon find how flat is the simple statement of the truth, and how natural — nay, how almost necessary it is, for the advertiser to tell "the truth, the whole truth and a good deal more than the truth." Combine this strong temptation with the security afforded by absolute secrecy as to the real nature of the article thus vaunted, and the necessary result is, the quack medicine literature of the present day. How can an honest man countenance such imposture? And how can any man claiming common sense, entrust his life and health and those of his family to the care of men whom he knows to be unscrupulous liars?

But after all, the one comprehensive reason why no intelligent or conscientious person should use them is that *they are secret*, and therefore cannot be intelligently used. They may be sometimes useful, sometimes useless, and sometimes injurious. To give a single example—a purgative pill, or a "Jaundice Bitters," whose virtues mainly depend upon aloes, may be very useful in a case requiring the peculiar action of that drug, but will be nothing less than a torment to a patient with any tendency to hemorrhoids. To a physician, or to any one who has thought on this subject, it seems self-evident that a remedy should be adapted to the nature and condition of the patient, and to the exact state of the disease. Yet I fear a majority even of the intelligent class who read the Bistoury, are accustomed to think that a medicine that is "good for a headache," or "good for a cold," in one case, must be equally good in every other case, and in every stage of the complaint. In other words, the theory of Specific Remedies, that relic of the charm-system of treatment, though confined by intelligent physicians to very narrow limits, or even denied *in toto*, is so convenient as a theory, that it very naturally remains popular among the community at large as a basis of treatment.

To sum up, then, most intelligent reader, you are urged to let these nostrums alone, because if you buy them you are cheated, because you thereby countenance and encourage a fraud upon the community, and because these articles, being secret, can never be used with safety.

---

Treatment of Headache. — Dr. Lauder Brunton, in a paper "On the Action of Purgative Medicines," recently published in the *Practitioner*, writes: "The administration of a brisk purgative, or small doses of Epsom salts, thrice a day, is a most effectual remedy for frontal headache when combined with constipation; but if the bowels be regular, the morbid processes on which it depends seem to be checked, and the headache removed even more effectually, by nitro-hydrochloric acid or by alkalies, given before meals. If the headache be immediately above the eyebrows, the acid is best; but if it be a little higher up, just where the hair begins, the alkalies appear to me to be the more effectual. At the same time that the headache is removed, the feelings of sleepiness and weariness, which frequently lead the patients to complain that they rise up more tired than they lie down, generally disappear."

